# Effect of Kinesio taping on Pregnancy-related low back pain: A protocol for systematic review and meta-analysis

**DOI:** 10.1371/journal.pone.0261766

**Published:** 2022-01-20

**Authors:** Xiali Xue, Xinwei Yang, Zhongyi Deng, Yan Chen, Xiaorong Mao, Huan Tu, Ling Zhou, Ning Li, Junzhi Sun, Ying He, Shuang Zhang

**Affiliations:** 1 Institute of Sports Medicine and Health, Chengdu Sport University, Chengdu, Sichuan, PR China; 2 School of Sports Medicine and Health, Chengdu Sport University, Chengdu, Sichuan, PR China; 3 Department of Gynecology and Obstetrics, Chengdu First People’s Hospital, Chengdu, Sichuan, PR China; 4 Key Laboratory of Obstetrics & Gynecologic and Pediatric Diseases and Birth Defects of Ministry of Education, Sichuan University West China Second University Hospital, Chengdu, Sichuan, PR China; 5 Nursing Research Center, University of Electronic Science and Technology of China Sichuan Provincial People’s Hospital, Chengdu, Sichuan, PR China; 6 Office of Academic Research, Chengdu Sport University, Chengdu, Sichuan, PR China; Istanbul University Istanbul Faculty of Medicine: Istanbul Universitesi Istanbul Tip Fakultesi, TURKEY

## Abstract

**Background:**

Pregnancy-related low back pain (PLBP) affects the daily living activities of pregnant women, even leading to fetal agitation and threatened abortion. Kinesio taping (KT) can improve the circulation of blood and provide elastic supports, which is a reliable method to treat low back pain. At present, although many studies have been published on the effects of KT on PLBP, the results are inconsistent, and some studies even report that KT does not affect PLBP. there is still a lack of high-level clinical evidence for the treatment of PLBP with KT. Therefore, this study proposes a protocol for a systematic review and meta-analysis of published Randomized Controlled Trials (RCTs) to evaluate the efficacy and safety of KT for PLBP.

**Methods:**

This protocol is guided by the Preferred Reporting Items for Systematic Reviews and Meta-Analyses Protocols. We will search the following database sources of the RCTs: PubMed, the Cochrane Library, EMBASE, Web of Science, Chinese Biomedical Literature Database (CBM), Chinese National Knowledge Infrastructure Database (CNKI), Chinese Science, and the Wanfang Database. From the establishment of the database to April 2021. The retrieval word adopts the combination of theme words and free words. Take “Kinesiotape, Tape Athletic, Orthotic Tape, Athletic Tape, Pregnancy, Pregnancies, Gestation, low back pain” as a term for retrieval. Two independent investigators will conduct an electronic literature search, study selection, data extraction, and quality assessment to summarize and evaluate the efficacy of KT in the treatment of PLBP. Retrospective trials are not included, and the risk of bias will be assessed using the Cochrane bias risk tool. All data analysis will be conducted using Revman5.3 software.

**Results:**

Quality outcomes in systematic review studies depend on inclusion and search criteria to obtain high-quality data, as well as how the data are processed and interpreted. Among the results, this study will objectively and comprehensively evaluate the efficacy and safety of the randomized controlled trial of KT in the treatment of PLBP, and make a detailed analysis of the effect of KT in the treatment of PLBP. The results will be analyzed by the Visual Analogue Scale of Pain and the Roland Morris Dysfunction Questionnaire. If applicable, a subgroup analysis will also be performed, which will be grouped according to the duration of pregnancy, grade of pain, etc. Finally, the results are submitted to a peer-reviewed journal for publication.

**Conclusion:**

Based on the results, this study will analyze and summarize the effect of KT on improving PLBP. It includes whether KT can improve the pain and lumbar function of PLBP, or it has adverse effects and reactions on pregnant women, then analysis and interpretation of other related issues. It is expected that the results of this study will provide a reference to the method and time of taping for clinical staff, as well as high-quality evidence to resolve the effect of KT on low back pain and provide corresponding guidance for pregnant women with low back pain. It aims to improve the status of low back pain in pregnant women and improve their physical health.

**Protocol registration number:**

PROSPERO CRD42021250373; https://clinicaltrials.gov/.

## 1. Introduction

Pregnancy-related low back pain (PLBP) is a common problem of pregnant women during pregnancy. It is a physiological pathology that only appears during pregnancy and postpartum. The etiology is not clear, but the main potential factors are hormones, biomechanics, post-traumatic or degenerative diseases, PLBP, and psychosocial factors [1]. Up to 70 percent of pregnant women have reported low back pain at some point during pregnancy [2,3]. With the increase of gestational age, the pain of pregnant women will be intensified. Which will affects their daily life, sleep, and so on, and it reduces the quality of life during pregnancy [4–7]. It also increases the risk of postpartum anxiety and depression [8]. Due to the lack of information on treatment options available to pregnant women and clinicians, and concerns that treatment may have harmful effects on fetal development, the treatment currently offered for PLBP is mainly physiotherapy [9].

Kinesio Taping (KT) is a non-invasive therapeutic technique developed by Dr. Kenzo Kase in 1973 [10]. Applied to patient skin under tension in the form of an elastic braid, it can be lengthways extended to 140 percent of its original length to treat a variety of musculoskeletal problems, such as injury, pain, dysfunction, and a variety of other conditions, without limiting joint mobility [11,12]. KT can adjust joint dislocation, provides muscle support, activates the endogenous analgesia system, and eliminates congestion and effusion [13,14]. KT can inhibit muscle overextension, relieve muscle spasticity and fatigue, reduce abnormal muscle spasticity, and enhance joint stability; Through sensorimotor stimulation, information is transmitted to the cerebral cortex, and then the motor response is generated; By stimulating the skin mechanoreceptors, improving the sensory feedback on the attached area, strengthening the input of proprioceptive sensory information, achieving indirect regulation of nerve and muscle activities, enhancing sensory input and other advantages [12,15].

With the continuous innovation and progress of rehabilitation methods, KT has been applied to improve PLBP. Some studies have shown that KT can reduce PLBP problems [16–18]. However, the evidence is still insufficient. Some studies have found that the improvement effect of KT on low back pain is not significant [19,20]. As RCTs are considered to be the highest quality evidence-based studies, this study will be carried out as RCT’s study. This study conducted a comprehensive evaluation of relevant research results from home and abroad through systematic evaluation and meta-analyze, to provide an evidence-based basis for the effect of KT on PLBP.

### 1.1. Objectives

The purpose of this study was to explore the efficacy and safety of KT in the treatment of PLBP and the possible mechanism of the effect of KT. It is expected to help clinicians select the appropriate treatment according to the specific condition of PLBP and suggest possible methods to prevent PLBP.

## 2. Methods

### 2.1. Study protocol registration

This systematic review protocol was registered with the International Prospective Register of Systematic Reviews (PROSPERO) (registration number: CRD42021250373). The consent of this protocol report is based on the preferred reporting items for systematic review and meta-analysis protocols (PRISMA-P) 2015 statement guidelines [21].

### 2.2 Inclusion criteria for study selection

#### 2.2.1 Types of studies

RCTs on the treatment of PLBP with KT were included in either Chinese or English, whether blind or assigned concealment was used.

#### 2.2.2 Types of patients

All patients met the diagnostic criteria of PLBP [22], and there was no clear classification of pain grade. The pain area was mainly between the 12th rib and the gluteal fracture area, but there could be radiating pain not exceeding the knee, which was often aggravated by a certain posture or activity. Pregnancy is between 14 and 40 weeks, no neurological symptoms or pregnancy complications. no recent history of medications, no previous back surgery, no serious back injury (spondylolisthesis), local tenderness, and unilateral or bilateral lumbar muscle tension.

#### 2.2.3 Type of intervention

The intervention measures for the experimental group were to receive KT intervention, while the control group received conventional rehabilitation training analgesics or placebo treatment. Inpatient Department (IPD) and interventions lasting more than two weeks and longer will be included. If the patient accepts KT and other treatments at the same time, it will not be considered. The way of sticking is to apply KT on both sides of the spine and abdomen to reduce the load of the lumbar spine and support abdominal muscles.

#### 2.2.4 Types of outcome measurements

Main outcome measures: Roland Morris Dysfunction Questionnaire (RMDQ); Secondary outcome measure: Visual Analogue Scale of pain (VAS).

#### 2.2.5 Inclusion criteria

This study will use the PICOS (Participants, Intervention, Comparator, Outcome, and Study design) model to select studies for this review.

1. Participants: patients with PLBP;
2. Intervention: patients received KT;
3. Comparator: patients received other treatment;
4. Outcomes: low back pain function and lumbar function improvement score;
5. Study design: Randomized clinical trial.

#### 2.2.6 Exclusion criteria

1. Non-Chinese and English literature;
2. Lack of outcome indicator data;
3. Duplicate studies, studies reporting too little information, studies with incomplete data.

### 2.3 Data sources

The following electronic databases will be searched from inception to April 2021: PubMed, the Cochrane Library, EMBASE, Web of Science, Chinese Biomedical Literature Database (CBM), Chinese National Knowledge Infrastructure Database (CNKI), Chinese Science, and the Wanfang Database. In addition, reference lists of the included studies were manually searched to identify additional relevant studies. This study will also search the following registration website of the clinical trial: WHO ICTRP and ISRCTN Register. Moreover, the relevant grey literature from the Health Management Information Database (HMIC), Open SIGLE Database, and the National Technical Information Service (NTIS) will be searched.

### 2.4 Search strategy

The search will be carried out by combining theme words with free words. The search terms on PubMed are as follows: Kinesio tape (e.g., Athletic Tape); Pregnancy (e.g., Pregnancies or Gestation); Randomized controlled trial (e.g., Randomized or Randomly). Combinations of Medical Subject Headings (MeSH) and text words will be used. The same search term is used in other electronic databases. Taking PubMed as an example, the retrieval strategy is shown in Table 1.

**Table 1:** Search strategy for the PubMed database.

Number	Search items
#1	Kinesiotape [MeSH]
#2	Kinesio Tape [Title/Abstract]
#3	Kinesio Tapes [Title/Abstract]
#4	Tape, Kinesio [Title/Abstract]
#5	Tapes, Kinesio [Title/Abstract]
#6	Athletic Tape [Title/Abstract]
#7	Tape, Athletic [Title/Abstract]
#8	Orthotic Tape [Title/Abstract]
#9	Tape, Orthotic [Title/Abstract]
#10	#1 OR #2 - #9
#11	Pregnancy [MeSH]
#12	Pregnancies [Title/Abstract]
#13	Gestation [Title/Abstract]
#14	#11 OR #12 - #13
#15	Randomized controlled trial [Publication Type]
#16	Randomized [Title/Abstract]
#17	Randomly [Title/Abstract]
#18	#15 OR #16 - #17
#19	#10 AND #14 AND #18

### 2.5 Data collection and analysis

#### 2.5.1 Selection of studies

**Fig 1:**
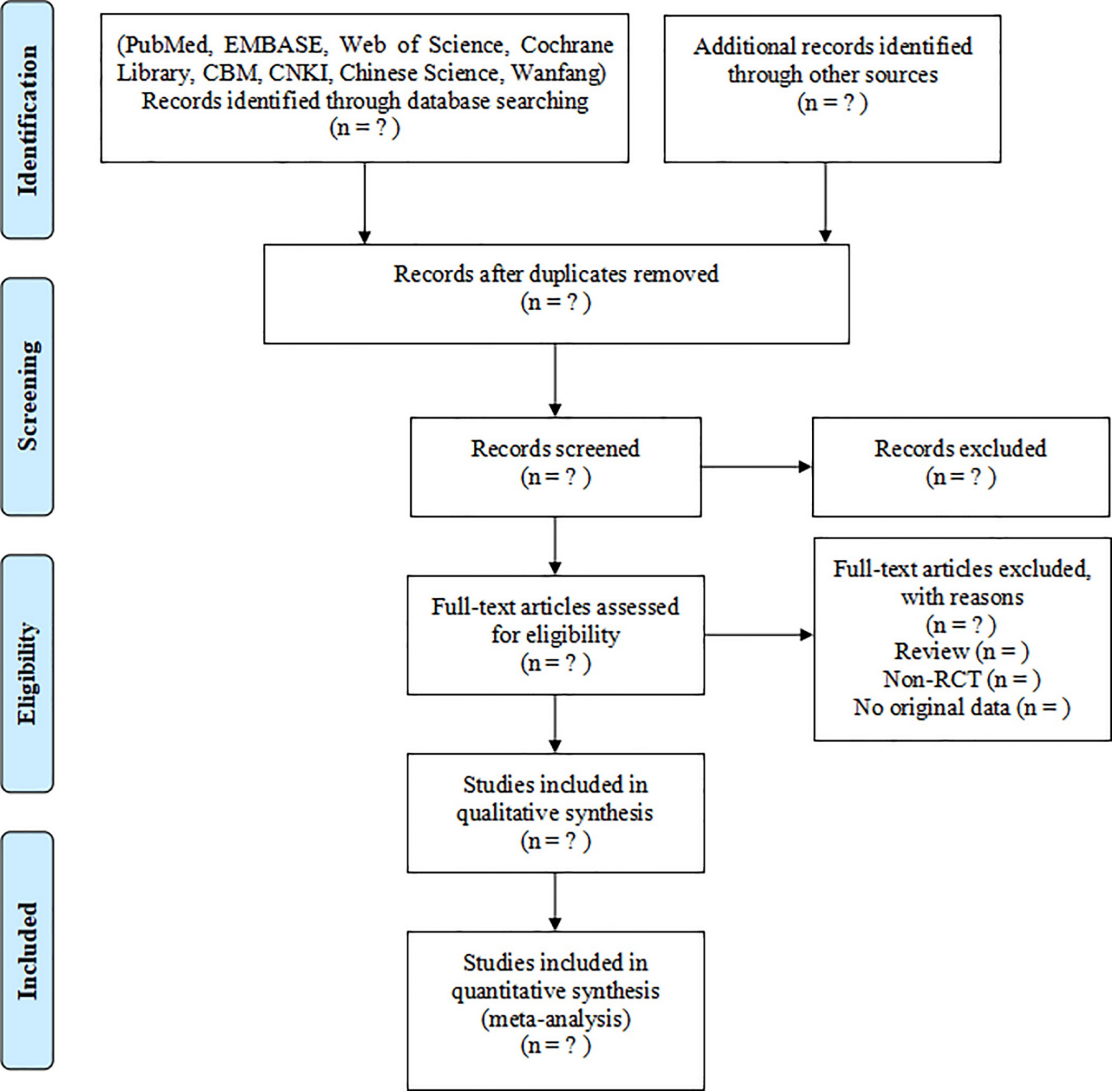
Flow chart of the study selection.

The retrieved studies will be imported into Endnote X8 to remove duplicates. Two researchers (XXL and CY) will independently screen the titles and abstracts according to the pre-established inclusion and exclusion criteria. After that, the full text will be screened as a second filtration. Two researchers will crosscheck the included studies, and the third researcher (LN) will be involved if disagreements occur. The literature screening flow chart is shown in Fig 1.

#### 2.5.2 Data extraction and management

Two researchers (TH and DZY) will independently extract the data and fill it in a predesigned form. Information includes author, year of publication, participant characteristics, details of interventions and comparisons, specific data, outcomes, conclusions, follow-up, adverse events, etc. The extracted data will be cross-checked. If there is a disagreement, consult a third researcher (LN) to reach a consensus. The study authors will be contacted for further information if necessary.

#### 2.5.3 Risk of bias in individual studies

Two researchers (MXR and ZL) independently evaluated the risk of bias of the included studies and cross-checked the results. Disagreements were resolved by consulting a third party. The quality of the included studies was assessed using the Cochrane Collaboration risk assessment tool for RCTs [23]. The risk of bias (low, unclear, or high) was assessed based on random sequence generation, allocation concealment, blinding of participants and personnel, blinding of outcome assessment, incomplete outcome data, selective reporting, and other biases.

#### 2.5.4 Management of missing data

When the data is missing, the first author or author of the article will be able to use the sensitivity analysis to assess the impact of the missing data on the results. If the effect is significant, we will remove the incomplete data experiment. After the data integrity is guaranteed, the descriptive analysis replaces the meta-analysis.

#### 2.5.5 Assessment of heterogeneity

Statistical heterogeneity will be investigated using tests and statistics. A fixed-effect model will be applied when heterogeneity is low (*I*^*2 *^< 50%) and a random-effects model will be used for moderate heterogeneity (50% <*I*^*2 *^< 75%). When heterogeneity is considerably high, meta-analysis will not be performed.

#### 2.5.6 Assessment of reporting biases

Funnel plots will be performed to assess potential reporting bias when more than ten studies are included. In addition, Egger regression and the Begg correlation test will be conducted to identify the funnel plot asymmetry.

#### 2.5.7 Assessment of reporting biases

This study will carry out funnel plots to evaluate and discuss any potential reporting bias, analyze the source of bias, and make a reasonable explanation.

#### 2.5.8 Subgroup analysis

When data are sufficient, this study will also perform subgroup analyses stratified by ethnicity (incidence of low back pain in pregnancy between different ethnic groups), smoking habits (incidence of low back pain in pregnancy between smokers and non-smokers), maternal use of painkillers, intervention cycle, pregnancy cycle, outcome indicators, and other details. Analyze the influence of different factors on PLBP to better explain the source of heterogeneity.

#### 2.5.9 Sensitivity analysis

Sensitivity analysis will be performed according to sample size, study design, heterogeneous quality, and statistical model. Trials with quality defects will be excluded to ensure the stability of the analysis results.

#### 2.5.10 Grading the quality of evidence

This study will use the evidence quality rating method to evaluate the results obtained from this analysis. GRADE will be assessed across the domains of risk of bias, consistency, directness, precision, and publication bias. In the context of the system review, quality reflects our confidence in the effectiveness of the assessment. It has four evaluation levels, namely, high (further research is very unlikely to change our confidence in the estimate of effect), moderate (further research is likely to have an important impact on our confidence in the estimate of effect and may change the estimate), low (further research is very likely to have an important impact on our confidence in the estimate of effect and is likely to change the estimate), or very low (very uncertain about the estimate of effect) [24].

### 2.6 Statistical analysis

All data were statistically analyzed using RevMan 5.3 software. Effect sizes for studies using the same result measurements were presented as the mean difference (MD) and the corresponding 95% confidence interval (CI). For studies using different outcome measures, the effect size was presented as the standardized mean difference (SMD), i.e., the change in the mean value of the selected outcome measure expressed as the weighted standard deviation mean difference. Statistical heterogeneity between the studies was evaluated with the *P* and *I*^*2*^ values, where P < 0.1 and *I*^*2*^ > 50% demonstrated high heterogeneity, and a random-effects model was used. A fixed-effects model was applied when the level of heterogeneity was not significant. The chi-square test was used to determine whether the combined statistics of several similar studies were significantly different. Subgroup analysis or meta-regression will be performed to assess the potential sources with reasonable explanations if heterogeneity is considerably high. Funnel plots will be employed to assess publications bias and if deemed relevant further statistical-based tests such as Egger’s test and Begg’s test, may be implemented. In addition, trim and fill methods will be implemented to test the robustness of the findings. The meta-analysis was set to a significant level of *P*<0.05. Finally, if a meta-analysis is not possible, due to a lack of quantitative information or if there is considerable heterogeneity between studies, a systematic review with descriptive analysis will be conducted.

### 2.7 Ethics and dissemination

The present study will use published data and does not require ethics approval.

## 3. Results

The results section mainly included the literature screening process, the number of RCTs and patients eventually included, the basic characteristics of the included studies, the risk assessment table of bias of the included studies, the specific results of Meta-analysis (forest map), and the test of publication bias. The results of the meta-analysis mainly included VAS score for pain improvement and RMDQ score for dysfunction improvement, according to the forest figure to interpret KT and relevant data on the effect of PLBP, at the same time will be under the relevant data of heterogeneity inspection and analysis, subgroup analysis if applicable, will be carried out under the method of subgroup analysis strategy subgroup analysis.

## 4. Discussion

As low back pain is a condition with high incidence and prevalence in the general population, PLBP is a common disease during pregnancy [25,26]. At present, the pathophysiological mechanisms associated with PLBP are unclear. The main accepted factors include weight gain during pregnancy, changes in postural position, and hormonal fluctuations that may cause musculoskeletal system problems, destabilizing the spine and sacroiliac joints, and connective tissue [27]. As a non-invasive form of treatment, KT is highly acceptable and harmless to the body. Therefore, it is of great significance and role to judge the efficacy and safety of KT in the intervention of PLBP [28].

The possible mechanisms of KT to improve PLBP include improving lower-back stability and increasing proprioception, to improve posture control [29,30]. KT can effectively fit the skin and apply pressure, increase the space under the skin or between the dermis and epidermis, promote subcutaneous blood circulation and lymphatic reflux, and speed up the healing of the injured site through its tension, thus helping to eliminate the substances causing pain; KT can also produce continuous neural sensory input to skin receptors, thereby relatively inhibiting the sensory input of pain and improving its ability to reduce mechanical stimulation of soft tissue during lumbar movement [31,32]. Pain relief is the most important evaluation indicator in treatment because the pain will seriously affect the daily life of pregnant women. The key to relieving pain with KT is how to choose the appropriate position, adjust the appropriate tension and determine the time of adhesive tape. In addition, in the United Kingdom and the United States, the treatment of PLBP usually includes health education on low back pain, starting from the first trimester of pregnancy, posture, and body mechanics education, such as the type of pillow to use when sleeping, and physical therapy.

Some studies have shown that KT can effectively reduce the symptoms of PLBP [9,17], but its efficacy has not been systematically evaluated scientifically. This study will systematically evaluate and meta-analyze the effect of KT on PLBP from four aspects of literature collection, literature screening, data extraction, and data analysis. It is expected that the results of this systematic review will help to improve the clinical evidence of KT in the treatment of PLBP and provide a basis for clinical decision-making.

However, this systematic review has some limitations. due to the limitation of language, we only searched Chinese and English literature. In the included studies, there may be differences in the shape, method, location, and length of KT taping in different studies, which may also lead to clinical heterogeneity.

## 5. Conclusion

It is the first time to evaluate the efficacy and safety of KT in the treatment of PLBP systematically. This study will draw relevant conclusions based on the efficacy of KT on PLBP, expecting to contribute to the prevention and treatment of PLBP, so to improve the health of pregnant women during pregnancy.

## Supporting information

S1 Checklist PRISMA-P (Preferred Reporting Items for Systematic Review and Meta-Analysis Protocols) 2015 checklist: Recommended items to address in a systematic review protocol. (ZIP)
